# Fabrication of PPy Nanosphere/rGO Composites via a Facile Self-Assembly Strategy for Durable Microwave Absorption

**DOI:** 10.3390/polym10090998

**Published:** 2018-09-06

**Authors:** Ying Wang, Yunchen Du, Bo Wu, Binhua Han, Shaoming Dong, Xijiang Han, Ping Xu

**Affiliations:** 1 MIIT Key Laboratory of Critical Materials Technology for New Energy Conversion and Storage, School of Chemistry and Chemical Engineering, Harbin Institute of Technology, Harbin 150001, China; wangying901115@hit.edu.cn (Y.W.); 11749060@mail.sustc.edu.cn (B.W.); BinHuaHan@yeah.net (B.H.); Dong_ShaoMing@163.com (S.D.); 2 Key Laboratory of Functional Inorganic Material Chemistry, Ministry of Education of the People’s Republic of China, Heilongjiang University, Harbin 150080, China

**Keywords:** polypyrrole nanospheres, reduced graphene oxide, microwave absorption, lightweight, durability

## Abstract

Traditional magnetic metal and alloy materials suffer from easy oxidation and high density, which hinders their practical application as high-performance microwave absorbers. Lightweight and durability have become new goals in the fabrication of the next generation of microwave absorbers. Herein, we report the synthesis of polypyrrole (PPy) nanosphere/reduced graphene oxide (rGO) composites through chemical reduction of self-assembly PPy nanosphere/GO hybrids. PPy nanospheres and GO are integrated effectively by π–π interaction of dual conjugated systems. When the mass ratio of PPy nanospheres to rGO is 0.6:1, the resultant composite, PPy/rGO-0.6, presents comparable/superior reflection loss characteristics to those magnetic metals and their related graphene-based composites in previous studies. Electromagnetic analysis reveals that well-matched characteristic impedance, multiple polarization loss, and good conductivity loss are, together, responsible for the excellent microwave absorption performance of PPy/rGO-0.6. More importantly, PPy/rGO-0.6 also exhibits good microwave absorption after being treated at 423 K for a long time. This work provides a new idea for designing and preparing a high-performance microwave absorber with lightweight and durable features.

## 1. Introduction

Due to the explosive development and popularization of communication devices and computer networks in the past decades, a large amount of electromagnetic (EM) waves have been released into the living environment of mankind, evolving into a serious EM pollution issue that is placing social development and human health in danger [1,2,3,4]. Numerous efforts have been devoted to developing microwave absorbers with a powerful absorption ability in a wide frequency band, to alleviate the negative effects of EM waves [5,6]. Traditionally, magnetic metals and their alloy nanopowders are one kind of the most promising candidates for high-performance microwave absorbers because of their compatible magnetic loss and dielectric loss, as well as high Snoek’s limit in gigahertz range [7]. For example, porous coin-like Fe [8], porous Co assemblies [9], FeCo nanoplates [10], CoNi microflowers [11], and granular FeCoNi films [12], have been confirmed to have strong magnetic loss characteristics and good microwave absorption. However, easy oxidation and high density of these magnetic materials have, so far, hindered their practical application to some extent. Therefore, developing novel and high-performance microwave absorbers with the features of being lightweight and durable still remains a challenge in the field of microwave absorption.

As a burgeoning member in the carbon family, reduced graphene oxide (rGO) has extremely large specific surface area and high carrier mobility, abundant defects, and numerous functional groups [13] and, thus, it has received much attention in the pursuit of advanced microwave absorbers [14,15]. However, pure rGO usually fails to achieve good characteristic impedance matching, owing to its high conductivity, and this will lead to the strong reflection of incident EM waves, rather than desirable absorption [14,15]. Recent progress in rGO-based composites has been greatly stimulated by the discoveries of a highly efficient synergistic effect between rGO and various incorporators. Many rGO-based composites, e.g., Co_3_O_4_/rGO [16], NiFe_2_O_4_/rGO [17], Fe_3_O_4_/rGO [18], CoNi/rGO [19], FeNi/rGO [20], MoS_2_/rGO [21], ZnO/rGO [22], CoS_2_/rGO [23], MnO_2_/rGO [24], and SiC/rGO [25], have been extensively developed as potential microwave absorbers. Although these rGO-based composites have made considerable achievements in microwave absorption performance, some of them with magnetic metals, alloys or ferrites are actually unable to be used persistently, due to their poor oxidation resistance, and others with dual dielectric media always require large absorber thickness to reach comparable microwave absorption performance to conventional magnetic metals/alloys. More recently, conjugated polymers, such as polyaniline (PANI), polypyrrole (PPy), and poly(3,4-ethylenedioxythioxythiophene) (PEDOT), have emerged as fascinating incorporators for novel microwave absorbers, due to their tunable electrical conductivity, profitable electron affinity, low density, ease of preparation, and good environmental stability [26,27,28]. For example, Yu et al. prepared PANI/rGO composite through orienting a perpendicular growth of PANI nanorods on the surface of rGO, and the resultant composite presented very strong reflection loss of −45.1 dB with the absorber thickness of only 2.5 mm [29]. Wu et al. reported that a self-assembled spongelike ultralight aerogel, consisting of PPy and rGO, could show an effective microwave absorption bandwidth (<−10 dB, 90% absorption) of 6.8 GHz (10.2–17.0 GHz) [30]. Zhang et al. decorated graphene sheets with PEDOT nanofibers, whose reflection loss could reach up to −48.1 dB at 10.5 GHz [31]. It is confirmed that the reinforced dielectric relaxation, special structural characteristics, and the charge transfer between conjugated polymer and rGO, are primarily responsible for the enhanced microwave absorption of these conjugated polymer/rGO composites. Among them, PPy/rGO composites are becoming the most typical representatives rendered by their diversified assembly modes and microstructures [32,33]. In our previous work, PPy nanospheres have displayed their own functions in optimizing the characteristic impedance and intensifying the reflection loss properties of PANI [34]. Therefore, the incorporation of rGO with PPy nanospheres may also have a great potential for microwave absorption.

Herein, we report the fabrication of PPy nanosphere/rGO composites via a facile self-assembly strategy and subsequent chemical reduction. The tedious functionalization steps are avoided due to the specific π–π interaction between PPy nanospheres and GO. Compared with many previous reports about PPy/graphene [30,32,33], the self-assembly strategy in our work has some unique advantages in controlling composition, saving time, and increasing yield. The as-prepared PPy nanosphere/rGO composites with different mass ratios are investigated in detail. The results indicate that the mass ratio of PPy nanospheres to rGO plays a crucial role in deciding the morphology, microstructure, and EM properties of the PPy nanosphere/rGO composites. With the optimized mass ratio, PPy nanospheres and rGO can be effectively integrated, leading to improved characteristic impedance matching. EM analysis reveals that both multiple polarization loss and conductivity loss account for the excellent microwave absorption of PPy nanosphere/rGO composites. It is very interesting that the low-density PPy nanosphere/rGO composite can display durable performance for microwave absorption application, which satisfies, well, the urgent demands for novel microwave absorbers in the future.

## 2. Materials and Methods

### 2.1. Materials Preparation

#### 2.1.1. Synthesis of PPy Nanospheres

PPy nanospheres were prepared as previously described [35]: first, 1.0 mL of pyrrole monomer and 0.1 g of FeCl_2_·4H_2_O were dissolved in 60 mL of ultrapure water. Then, 5.0 mL of H_2_O_2_ was introduced to trigger the polymerization of pyrrole monomer. The mixture was continuously stirred at room temperature for 8 h. The dark PPy nanospheres were collected by centrifugation, washed with acetone several times, and finally dried at 60 °C for 12 h.

#### 2.1.2. Synthesis of PPy Nanosphere/rGO Composites

Commercial graphene oxide (GO) (0.05 g) was dispersed in 50 mL of ultrapure water under ultrasonic treatment for 2 h to form suspension A. The required amounts of PPy nanospheres were dispersed in 50 mL of ultrapure water under magnetic stirring for 30 min to form suspension B. Suspension B was added into suspension A, and then the mixture was slowly stirred for another 30 min to complete the interfacial interactions between PPy nanospheres and GO. To obtain PPy nanosphere/rGO composites, 25 μL of N_2_H_4_·H_2_O (80 wt %) as reductant was added, dropwise, into the mixture of PPy nanospheres and GO. The composites were finally obtained by evaporating the excess water at 80 °C, and were denoted as PPy/rGO-S1, PPy/rGO-S2, and PPy/rGO-S3, whose mass ratios of PPy nanospheres to rGO corresponded to 1.2:1, 0.6:1, and 0.3:1, respectively. For comparison, pure rGO was fabricated by using the same procedures in the absence of PPy nanospheres.

### 2.2. Physical Characterization

Zeta potential analyzer (Malvern Zetasizer Nano Z, Malvern Instruments, Malvern, UK) was used to measure zeta potentials. The pH values were determined by pH meter (PHS-3E PH Meter, Shanghai Precision & Scientific Instrument, Shanghai, China). Scanning electron microscope (SEM) images were obtained on a Hitachi S-4300 (Hitachi, Tokyo, Japan). Transmission electron microscope (TEM) images and high-resolution TEM (HR-TEM) were obtained on a JEM-3000F (JEOL, Tokyo, Japan). Raman spectra were measured on a confocal Raman spectroscopic system (In Via, Renishaw, Gloucestershire, UK) using a 532 nm laser. X-ray photoelectron spectra (XPS) were obtained with a PHI 5700 ESCA system (Physical Electronics, Chanhassen, MN, USA) equipped with an A1 Kα radiation as the source (1486.6 eV). An Agilent N5230A vector network analyzer (Agilent, Palo Alto, CA, USA) was used to determine the complex permittivity and complex permeability in the frequency range of 2.0–18.0 GHz, for calculating the reflection loss. A sample containing 30 wt % of as-prepared product was pressed into a ring with an outer diameter of 7 mm, an inner diameter of 3 mm, and a thickness of 2 mm for microwave measurement, in which paraffin wax was used as the binder.

## 3. Results and Discussion

**Figure 1:**
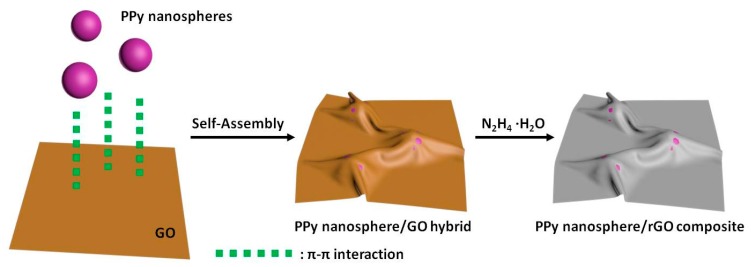
Schematic illustration of preparing polypyrrole (PPy) nanosphere/reduced graphene oxide (rGO) composite.

**Figure 2:**
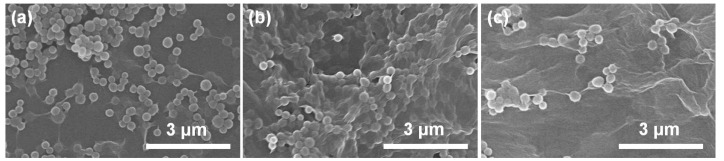
SEM images of PPy/rGO-S1 (**a**), PPy/rGO-S2 (**b**), and PPy/rGO-S3 (**c**).

**Figure 3:**
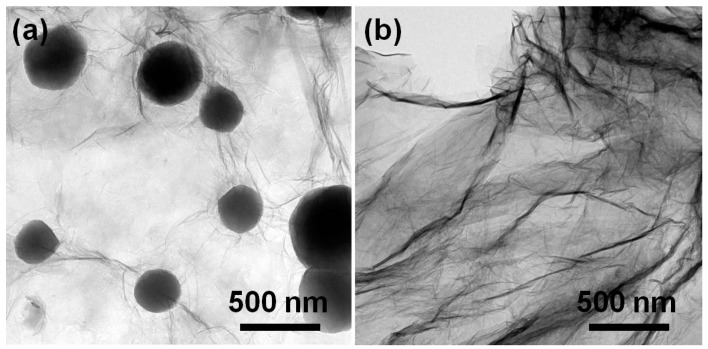
TEM images of PPy/rGO-S2 (**a**) and rGO (**b**).

In general, the self-assembly of conjugated polymers and GO can be driven by the electrostatic interaction and π–π interaction arising from dual conjugated systems [36,37]. Zeta potential is a physical parameter that can characterize the surface charge of nanomaterials, and the electrostatic interaction only occurs between different nanomaterials with reverse zeta potentials [38]. By considering that zeta potentials are always pH-dependent, we primarily measure the pH values of PPy nanosphere suspension (0.6 mg/mL) and GO suspension (1.0 mg/mL). As shown in Table S1, the pH values of PPy nanosphere and GO suspensions are 6.62 and 2.35, and their corresponding zeta potentials are −42.4 and −35.0 mV, respectively. These results indicate that both PPy nanosphere and GO are negatively charged in their suspensions. Even if we adjust the pH value of PPy nanosphere suspension to 2.35, the zeta potential is still −18.2 mV, which means that the electrostatic interaction will not contribute to the self-assembly process between PPy nanosphere and GO. By contrast, XPS spectra not only confirm the presence of PPy nanosphere in the PPy nanosphere/GO hybrid through discerning the signal of the C–N bond, but also reveal a slight shift of the binding energy of N–H bond from 399.3 to 399.8 eV (Figure S1). These results suggest dual π-conjugated systems induce the strong interaction between PPy nanosphere and GO [39]. Moreover, one can find that the PPy nanosphere suspension and GO suspension are very stable (Figure S2a,b), and the PPy nanosphere/rGO composite inherits the colloidal stability from its individual components, even after static treatment for 48 h since the PPy nanosphere/GO is reduced by N_2_H_4_·H_2_O (Figure S2c). However, when pure GO suspension is reduced by N_2_H_4_·H_2_O, the resultant rGO will sedimentate in a short time (Figure S2d), which is a hint that the self-assembly of PPy nanospheres and rGO can suppress the aggregation of rGO effectively. In view of these facts, the strategy for preparing PPy nanosphere/rGO composites can be schematically depicted in Figure 1, where PPy nanospheres and GO are integrated together through π–π interactions, and then the PPy nanosphere/GO hybrid is transformed into PPy nanosphere/rGO composite by chemical reduction. The morphology and microstructure of the as-prepared PPy nanospheres, rGO, and PPy nanosphere/rGO composites are investigated by SEM. As shown in Figure S3, PPy nanospheres display smooth surfaces and an average diameter of 330 nm in terms of the statistical data of diameter. For PPy/rGO-S1 (Figure 2a), it is clear that some PPy nanospheres with loose dispersion are concealed under rGO, and that some other PPy nanospheres aggregate on the external surface of rGO, which probably results from excess PPy nanospheres that hinder the self-assembly between PPy nanospheres and GO. When the mass ratio of PPy nanospheres to rGO is properly decreased, PPy nanospheres with good dispersion are totally covered by rGO in PPy/rGO-S2 (Figure 2b), implying full contact between PPy nanospheres and GO through π–π interactions. However, a lower mass ratio may reduce the relative content of PPy nanospheres greatly, and these nanospheres with loose dispersion are completely coated by rGO (Figure 2c). The morphology and microstructure of PPy/rGO-S2 are further studied by TEM in Figure 3a. As observed, PPy nanospheres are wrapped in rGO, and they link with each other by rGO as a medium. The visible wrinkles of rGO on the edge of PPy nanospheres imply the strong affinity between PPy nanospheres and rGO, which may generate considerable interfacial effects and be quite beneficial to enhancing the dielectric loss ability of PPy nanosphere/rGO composites [30,40]. In addition, one can find that pristine GO sheets are fully outstretched (Figure S4a), while there is serious crimp and aggregation in pure rGO, due to the strong π–π restacking after removing the surface functional groups (Figure 3b and Figure S4b). Compared with pure rGO, there is less crimp and aggregation in PPy/rGO-S2 suggesting that PPy nanospheres can act as obstructions to impede the restacking of rGO [41,42].

**Figure 4:**
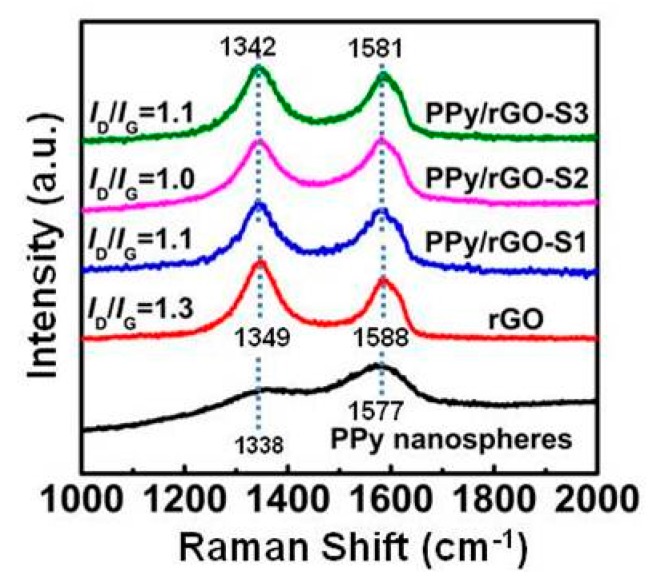
Raman spectra of PPy nanospheres, rGO, and PPy nanosphere/rGO composites.

Raman spectra of PPy nanospheres, rGO, and PPy nanosphere/rGO composites are depicted in Figure 4. The characteristic bands of pure PPy nanospheres are visible at 1338 cm^−1^ and 1577 cm^−1^, due to the ring stretching mode and the stretching of the C=C backbone, respectively [43]. By comparison, two peaks at 1349 cm^−1^ (D-band) and 1588 cm^−1^ (G-band) are also observed in the Raman spectra of rGO, where the D-band represents the defects and disorder in rGO, and the G-band indicates its graphitic component [44]. It is found that D-bands and G-bands with similar profiles can be easily distinguished in all PPy nanosphere/rGO composites, because PPy nanospheres cannot cover rGO completely, and there are still quite a few rGO sheets exposed to the laser irradiation of Raman spectra (Figure 2). Compared with pure rGO, the slight blueshifts for both D-band and G-band in PPy nanosphere/rGO composites reveal charge redistribution and coupling between PPy nanospheres and rGO [45]. Although the mass ratios of PPy nanospheres to rGO are completely different, these PPy nanosphere/rGO composites exhibit almost identical *I*_D_/*I*_G_ values, which demonstrates the highly effective hybridization between PPy nanospheres and rGO. Meanwhile, the *I*_D_/*I*_G_ values of PPy nanosphere/rGO composites are lower than that of pure rGO, because the wrinkles of pure rGO, known as physical defects, contribute to the increased *I*_D_/*I*_G_ value [46]. As mentioned above, rGO usually suffers from severe bending and aggregation towards a partial graphitic structure, owing to strong π–π re-stacking [47], and this will result in high conductivity and unfavorable characteristic impedance mismatching. Both TEM images and Raman data suggest that the introduction of PPy nanospheres can effectively suppress the self-bending and self-aggregation of rGO, which will make for the matched characteristic impedance of PPy nanosphere/rGO composites.

**Figure 5:**
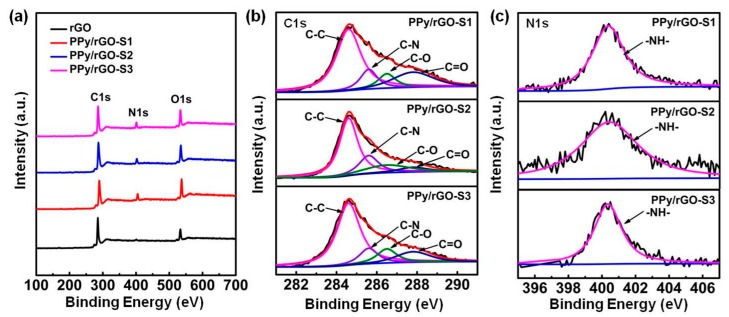
XPS wide-scan spectra of rGO, PPy/rGO-S1, PPy/rGO-S2, and PPy/rGO-S3 (**a**); C1s core-level spectra of PPy/rGO-S1, PPy/rGO-S2, and PPy/rGO-S3 (**b**); N1s core-level spectra of PPy/rGO-S1, PPy/rGO-S2, and PPy/rGO-S3 (**c**).

XPS measurements are further performed to investigate the elemental composition and bonding configurations of these PPy nanosphere/rGO composites. As shown in Figure 5a, the spectra show strong signals of C and O elements for rGO, and strong signals of C, N, and O for PPy nanosphere/rGO composites, indicating the successful incorporation of PPy nanospheres into rGO matrix. Both pure rGO and PPy nanosphere/rGO composites show significant decreases in the relative intensity of O element because of the removal of oxygen-containing functional groups by N_2_H_4_·H_2_O (Figure 5b). There is only one peak at 400.3 eV in N1s core-level spectra for all PPy nanosphere/rGO composites (Figure 5c) and the disappearance of –N^+^H– in PPy nanosphere/rGO composite is due to the fact that rGO can act as an electron donor for PPy nanospheres and, thus, lead to further hybridization between them. It is worth noting that when PPy nanosphere/GO hybrid is transformed into PPy nanosphere/rGO composite, the binding energy of the N–H bond will be further shifted from 399.8 to 400.3 eV by the enhanced π–π interaction between PPy nanospheres and rGO [48]. 

From the transmission line theory, the values of reflection loss (*RL*) can be calculated by the following equations [49]:

(1) $$RL\left(\mathrm{dB}\right)=20\log|\frac{Z_{\mathrm{in}}-1}{Z_{\mathrm{in}}+1}|,$$

**Figure 6:**
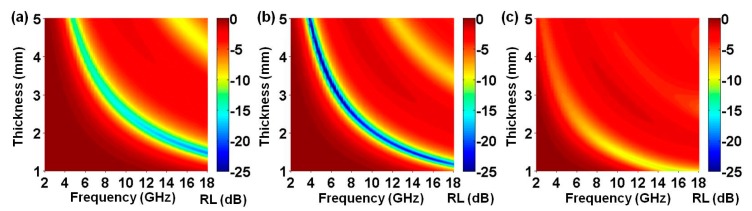
Reflection loss properties of PPy/rGO-S1 (**a**), PPy/rGO-S2 (**b**), and PPy/rGO-S3 (**c**).

*Z*_in_ is the normalized input impedance of a metal-backed microwave absorption layer and is deduced by [49],

(2) $$Z_{\mathrm{in}}=\sqrt{\frac{\mu }{\varepsilon }}\tan h\left[j\left(\frac{2\pi }{c}\right)fd\sqrt{\mu \varepsilon }\right],$$

where *ε* and *μ* are the complex permittivity and complex permeability of the microwave absorber, respectively, *f* is the microwave frequency, *c* is the velocity of EM waves in free space, and *d* is the thickness of an absorber. Based on these two equations, the frequency-dependent *RL* values of PPy nanosphere/rGO composites are plotted with varied absorber thickness (*d*), from 1.0–5.0 mm (the *RL* values are artificially adjusted to −25.0 dB for clarity). As shown in Figure 6, all PPy nanosphere/rGO composites can work for the consumption of incident EM waves, but their actual performances are distinguishable and very sensitive to the mass ratios of PPy nanospheres to rGO. The minimum *RL* values for PPy/rGO-S1, PPy/rGO-S2, and PPy/rGO-S3 are −19.0 dB at 18.0 GHz (Figure S5a), −59.2 dB at 5.0 GHz (Figure S5b), and −10.9 dB at 16.0 GHz (Figure S5c), respectively, and the corresponding effective bandwidths over −10 dB (90% absorption) of these PPy nanosphere/rGO composites are 13.8 GHz (4.2–18.0 GHz), 14.7 GHz (3.3–18.0 GHz), and 6.4 GHz (11.6–18.0 GHz), respectively. It is clear that the microwave absorption properties of PPy/rGO-S2 are significantly superior to the PPy/rGO-S1 and PPy/rGO-S3. In Table 1, we also list the *RL* properties of some magnetic metals, magnetic metal/graphene, magnetic metal/conjugated polymer, and conjugated polymer/graphene absorbers, and it can be seen that, even in the case of relative low loading, PPy/rGO-S2, in our work, can produce superior/comparable microwave absorption performance to most of these examples. These comparisons demonstrate that low-density PPy/rGO-S2 may be a better choice for microwave absorption application.

**Figure 7:**
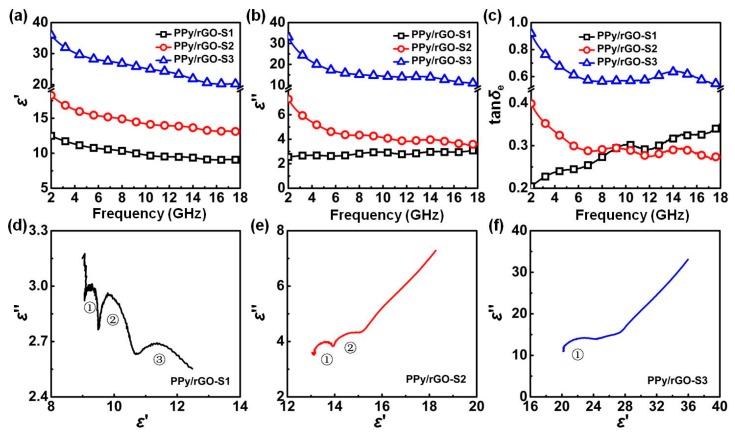
Real parts (**a**) and imaginary parts (**b**) of the complex permittivity of PPy/rGO-S1, PPy/rGO-S2, and PPy/rGO-S3, and their corresponding dielectric dissipation factor (**c**) in the frequency range of 2.0–18.0 GHz; typical Cole–Cole semicircles (*ε*″ vs. *ε*′) for PPy/rGO-S1 (**d**), PPy/rGO-S2 (**e**), and PPy/rGO-S3 (**f**).

It is widely accepted that microwave absorption properties of a microwave absorber are closely related to the complex permittivity (*ε* = *ε*′ − j*ε*″) and complex permeability (*μ* = *μ*′ − j*μ*″), where the real parts of complex permittivity (*ε*′) and complex permeability (*μ*′) represent the storage capabilities of electric and magnetic energy, and imaginary parts (*ε*″ and *μ*″) are associated with the loss capabilities of electric and magnetic energy, respectively [61]. As observed in Figure 7a,b, *ε*′ values of all PPy nanosphere/rGO composites display typical frequency-dependent behaviors, and they gradually decrease when the frequency increases. The curves of *ε*″ values of PPy/rGO-S1 and PPy/rGO-S2 present similar variation trends, while *ε*″ values of PPy/rGO-S1 shows a slight upward trend in the studied frequency range. In terms of Debye theory, *ε*′ and *ε*″ can be described as [3]

(3) $$\varepsilon^{\prime }=\varepsilon_{\infty }+\frac{\varepsilon_{s}-\varepsilon_{\infty }}{1+\omega^{2}\tau^{2}},$$

(4) $$\varepsilon^{\prime \prime }=\frac{\varepsilon_{s}-\varepsilon_{\infty }}{1+\omega^{2}\tau^{2}}\omega \tau +\frac{\sigma_{\mathrm{ac}}}{\omega \varepsilon_{0}},\;$$

where *ε*_s_ is the static permittivity, *ε*_∞_ is the relative dielectric permittivity at the high-frequency limit, *ω* is angular frequency, *τ* is polarization relaxation time, *σ*_ac_ is the alternative conductivity, and *ε*_0_ is the dielectric constant in vacuum (*ε*_0_ = 8.85 × 10^−12^ F·m^−1^). From Equation (3), it can be assured that the decrease of *ε*′ is attributed to the increase of *ω* in the studied frequency range. This phenomenon can be considered as dipole orientation polarization [62]. In addition, interfacial polarization is another important factor that is responsible for dielectric loss in nanohybrids or nanocomposites [63]. From PPy/rGO-S1 to PPy/rGO-S3, significant enhancements in both *ε*′ and ε″ are observed. As revealed in our previous report [34], PPy nanospheres have poor conductivity due to their low polymerization degree, which means that rGO will dominate the conductivity of these composites. Considering the free electron theory [64], $\varepsilon^{\prime \prime }\approx 1/2\pi \rho f\varepsilon_{0}$, where *ρ* is the resistivity, high conductivity (i.e., low resistivity) will be beneficial to enhancing the imaginary parts of complex permittivity. Thus, the increase of ε″ from PPy/rGO-S1 to PPy/rGO-S3 comes from the incremental ratios of rGO in these composites. Dielectric dissipation factor (tan*δ*_e_ = *ε*″/*ε*′) and magnetic dissipation factor (tan*δ*_m_ = *μ*″/*μ*′), are widely utilized to evaluate the dielectric loss and magnetic loss abilities of an absorber [61]. The tan*δ*_e_ of PPy/rGO-S3 is larger than that of PPy/rGO-S1 and PPy/rGO-S2 in the whole range from 2.0 to 18.0 GHz, while the tan*δ*_e_ of PPy/rGO-S2 is larger than that of PPy/rGO-S1 at 2.0–9.2 GHz, and is smaller than that of PPy/rGO-S1 at 9.2–18.0 GHz. In order to study polarization relaxation, the relationship between *ε*′ and *ε*″ without the second part of Equation (4) can be deduced as

(5) $${\left(\;\varepsilon \;\prime -\frac{\varepsilon_{s}-\varepsilon_{\infty }}{2}\right)}^{2}-{\varepsilon \prime \prime \;}^{2}={\left(\frac{\varepsilon_{s}-\varepsilon_{\infty }}{2}\right)}^{2}.\;$$

**Figure 8:**
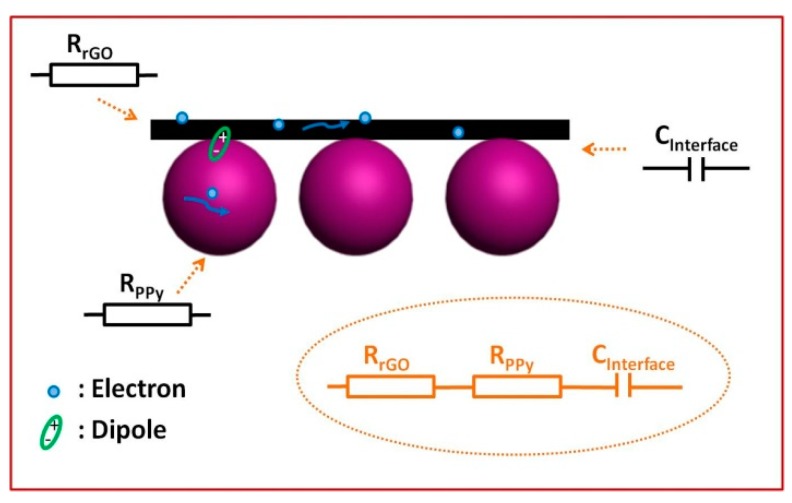
The model for the formation of conductive connections.

Each semicircle in the plot of *ε*″ versus *ε*’ may correspond to one Debye relaxation process [65]. As shown in Figure 7d–f, three semicircles, two semicircles, and one semicircle are found in PPy/rGO-S1, PPy/rGO-S2, and PPy/rGO-S3, respectively. The gradual decrease of semicircles in these composites suggests that Debye relaxation contribution is gradually weakened, and conductivity loss is becoming dominant. The corresponding conductive interconnections model has been shown in Figure 8. Specifically, the extended π-conjugated systems in both PPy nanosphere and rGO act as microresistances, and numerous capacitor-like junctions are formed between PPy nanospheres and rGO, owing to the accumulation and uneven distribution of space charges at their interfaces. In addition, it can be found that all PPy nanosphere/rGO composites are unable to generate any magnetic loss because of the absence of magnetic components, and thus, their values of *μ*′ and *μ*″ and corresponding magnetic dissipation factors are approximate constants, and close to 1, 0, and 0, respectively (Figure S6). Although PPy/rGO-S3 as dielectric material has superior dielectric loss ability, its microwave absorption performance is inferior. Apart from the attenuation characteristics, impedance matching has to be considered, because it only occurs when the complex permittivity and permeability values are close [66,67]. The gap between the complex permittivity and complex permeability of PPy/rGO-S3 is obviously larger than those of PPy/rGO-S1 and PPy/rGO-S2. Therefore, it can be inferred that the limited microwave absorption of PPy/rGO-S3 is attributed to its poor characteristic impedance matching. Compared with PPy/rGO-S1, PPy nanospheres with good dispersion are totally covered by rGO in PPy/rGO-S2, which creates abundant heterogeneous interfaces, and produces significant interfacial polarization (space charge polarization), resulting in the significantly enhanced microwave absorption performance.

**Figure 9:**
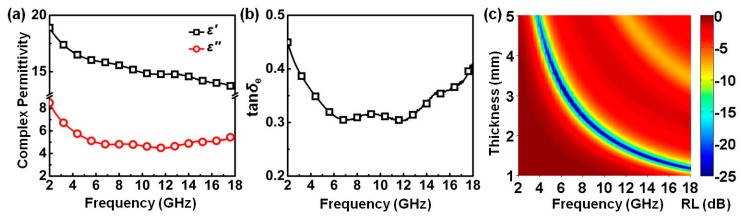
Complex permittivity (**a**), dielectric dissipation factor (**b**), and reflection loss properties of PPy/rGO-S2-423 K in the frequency range of 2.0 to 18.0 GHz (**c**).

As mentioned above, traditional magnetic metal and alloy materials usually suffer from severe performance degradation after being used for a period of time, due to their poor oxidation resistance. To test the durable performance of PPy nanosphere/rGO composite, PPy/rGO-S2 was treated at 423 K for 10 days, and the product is denoted as PPy/rGO-S2-423 K. Figure 9 shows the complex permittivity, dielectric dissipation factor, and reflection loss properties of PPy/rGO-S2-423 K. Compared with untreated PPy/rGO-S2, PPy/rGO-S2-423 K displays a slight increase in both *ε*′ and *ε*″, which may be associated with the electron hopping due to heat treatment. Meanwhile, the corresponding dielectric dissipation factor also shows a slight increase, indicating that the dielectric loss ability of PPy/rGO-S2 even gains a bit of enhancement after the heat treatment. By calculating the *RL* properties, it can be found that PPy/rGO-S2-423 K almost keeps identical microwave absorption performance, whose minimum values and effective bandwidths are −60.6 dB at 6.4 GHz and 14.7 GHz (3.3–18.0 GHz), respectively. These results forcefully validate the good durability of PPy/rGO-S2 as a microwave absorber. It is unavoidable that long-time ultraviolet irradiation can also induce the degradation of PPy chains, and result in the weakened microwave absorption performance of PPy/rGO-S2 [68], while this novel microwave absorber may still have wide application prospects in some specific areas (e.g., microwave dark room) that are not exposed to ultraviolet irradiation.

## 4. Conclusions

PPy nanosphere/rGO composites have been successfully prepared through a self-assembly strategy on basis of π–π interactions between PPy nanospheres and GO, and subsequent reduction by N_2_H_4_·H_2_O. The integrations of PPy nanospheres and rGO with different mass ratios have been investigated in detail. PPy nanospheres and rGO can be fully coupled under the optimum mass ratio and generate abundant heterogeneous interfaces. Well-matched characteristic impedance, in cooperation with multiple polarization loss and conductivity loss, accounts for the enhanced microwave absorption of PPy nanosphere/rGO composite. The superiority of PPy nanosphere/rGO composite is also addressed by a comprehensive comparison with some magnetic metals, magnetic metal/graphene, magnetic metal/conjugated polymer, and conjugated polymer/graphene absorbers. Additionally, the PPy nanosphere/rGO composite exhibits durable performance after a long-time thermal treatment. This work not only provides a way to rationally design lightweight, durable, and high-performance microwave absorbers, but also advances the understanding of microwave absorption mechanism for graphene-based composites.

## Figures and Tables

**Table 1:** Microwave absorption performance of some magnetic metals, magnetic metal/graphene, magnetic metal/conjugated polymer, and conjugated polymer/graphene absorbers in previous references and this work.

Absorber	Loading (%)	Integrated Thickness (mm)	*RL*_min_ (dB) (Frequency, Thickness)	Bandwidth over −10 dB (GHz)	Effective Bandwidth (*RL* < −10 dB) (GHz)	Ref.
PANI nanorods/graphene	50	2.0–5.0	−51.1 dB (6.4 GHz, 3.0 mm)	5.8–18.0	12.2	[29]
Flower-like α-Fe particles	40	2.0–5.0	−33.1 dB (17.5 GHz, 5.5 mm)	6.0–18.0	12.0	[50]
Hollow Co nanoparticles	70	1.1–3.0	−45.06 dB (8.0 GHz, 1.7 mm)	5.2–16.5	11.3	[51]
Fe nanoparticles	50	1.5–5.0	−33 dB (1.3 GHz, 5.0 mm)	0.9–3.7	2.8	[52]
Ni-10000	75	1.0–5.0	−17.9 dB (17.8 GHz, 1.2 mm)	4.2–18.0	13.8	[53]
Flower-like Ni	33	2.0–5.0	−17.0 dB (13.0 GHz, 3.0 mm)	11.5–14.0	2.5	[54]
Graphene–Ni composite	30	2.0–4.0	−42.0 dB (17.6 GHz, 2 mm)	8.0–18.0	10	[55]
α-Co/graphene	60	1.0–5.0	−47.5 dB (11.9 GHz, 2 mm)	3.0–15.0	12.0	[56]
FeCo/graphene	50	1.5–6.0	−40.2 dB (8.9 GHz, 2.5 mm)	3.4–18.0	14.6	[57]
Ni/PANI	50	2.0–6.0	−35.0 dB (17.2 GHz, 5.0 mm)	4.0–18.0	14.0	[58]
PANI/rGO	50	1.5–3.5	−41.4 dB (13.8 GHz, 2.0 mm)	6.0–18.0	12.0	[59]
PEDOT/rGO	10	2.0–4.0	−35.5 dB (13.3 GHz, 2.0 mm)	4.8–16.2	11.4	[60]
PPy/rGO-S2	30	1.0–5.0	−59.2 dB (5.0 GHz, 3.8 mm)	3.3–18.0	14.7	This work

## Supplementary Materials

The following are available online at http://www.mdpi.com/2073-4360/10/9/998/s1, Figure S1: XPS wide-scan spectra of GO, PPy nanospheres, and PPy nanosphere/GO hybrid (a); C1s core-level spectra of GO, PPy nanospheres, and PPy nanosphere/GO hybrid (b); N1s core-level spectra of PPy nanospheres and PPy nanosphere/GO hybrid (c); Figure S2: Digital photograph of commercial GO suspension (a), PPy nanosphere suspension (b), PPy nanosphere/rGO composite (mass ratio of PPy nanospheres and rGO is 0.6) suspension (c), and pure rGO suspension, after static treatment for 48 h; Figure S3: SEM image of PPy nanospheres, and the inset is the corresponding statistical data on the diameters of PPy nanospheres; Figure S4: SEM images of commercial GO (a) and as-prepared rGO (b); Figure S5: *RL* values of PPy/rGO-S1, PPy/rGO-S2, PPy/rGO-S3 with absorber thicknesses of 1.46 mm, 3.82 mm, and 1.00 mm, respectively; Figure S6: Real parts *μ*′ (a) and imaginary parts *μ*″ (b) of complex permeability of PPy/rGO-S1, PPy/rGO-S2, and PPy/rGO-S3, and their corresponding magnetic dissipation factor (c) in the frequency range of 2.0–18.0 GHz. Table S1: The pH values and zeta potentials of the aqueous dispersions of commercial GO and PPy nanosphere.
