# Supplementary material for: Fabrication of PPy Nanosphere/rGO Composites via a Facile Self-Assembly Strategy for Durable Microwave Absorption
Source: Polymers (Basel). 2018 Sep 6;10(9):998. doi: 10.3390/polym10090998 (PMC6403787; doi:10.3390/polym10090998)
Supplement: Supplementary file 1 [file polymers-10-00998-s001.pdf]

polymers

MDPI

Supplementary Information

Fabrication of PPy Nanosphere/rGO Composites via a Facile Self-Assembly Strategy for Durable Microwave Absorption

Ying Wang [1], Yunchen Du [1,2,*], Bo Wu [1], Binhua Han [1], Shaoming Dong [1], Xijiang Han [1,*] and Ping Xu [1,*]

1 MIIT Key Laboratory of Critical Materials Technology for New Energy Conversion and Storage, School of Chemistry and Chemical Engineering, Harbin Institute of Technology, Harbin 150001, China; wangying901115@hit.edu.cn (Y.W.); 11749060@mail.sustc.edu.cn (B.W.); BinHuaHan@yeah.net (B.H.); Dong_ShaoMing@163.com (S.D.)

2 Key Laboratory of Functional Inorganic Material Chemistry, Ministry of Education of the People's Republic of China, Heilongjiang University, Harbin 150080, China

* Correspondence: yunchendu@hit.edu.cn (Y.D.); hanxijiang@hit.edu.cn (X.H.); pxu@hit.edu.cn (P.X.); Tel.: +86-451-8641-8750 (X.H.)

Table S1. The pH values and zeta potentials of the aqueous dispersions of commercial GO and PPy nanosphere.

Samples	Concentration (mg/mL)	pH	Zeta potential (mV)
Commercial GO	1.0	2.35	−35.0
PPy nanosphere	0.6	6.62	−42.4
PPy nanosphere-HCl [1]	0.6	2.35	−18.2

[1] PPy nanosphere-HCl is the dispersion of PPy nanosphere whose pH value is adjusted to 2.35 by HCl.

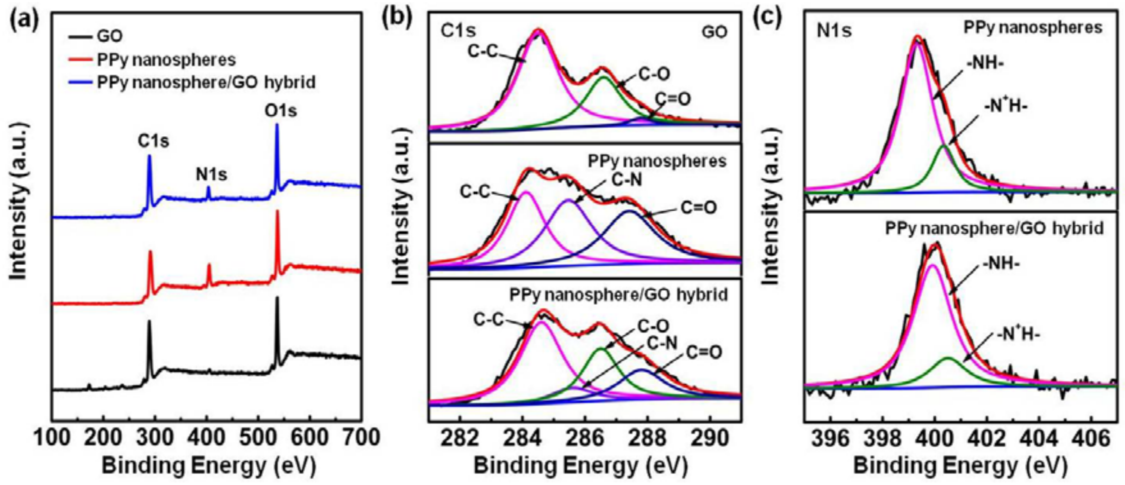

Figure S1. XPS wide-scan spectra of GO, PPy nanospheres, and PPy nanosphere/GO hybrid (a); C1s core-level spectra of GO, PPy nanospheres, and PPy nanosphere/GO hybrid (b); N1s core-level spectra of PPy nanospheres and PPy nanosphere/GO hybrid (c).

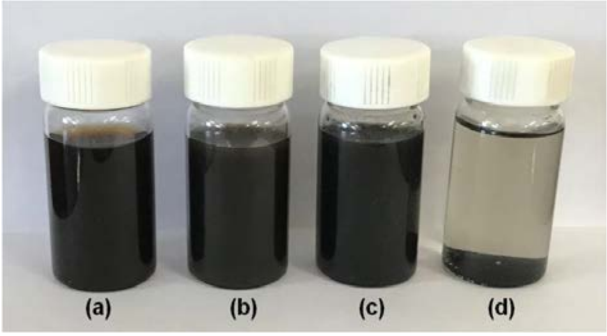

Figure S2. Digital photograph of commercial GO suspension (a), PPy nanosphere suspension (b), PPy nanosphere/rGO composite (mass ratio of PPy nanospheres and rGO is 0.6) suspension (c), and pure rGO suspension, after static treatment for 48 h.

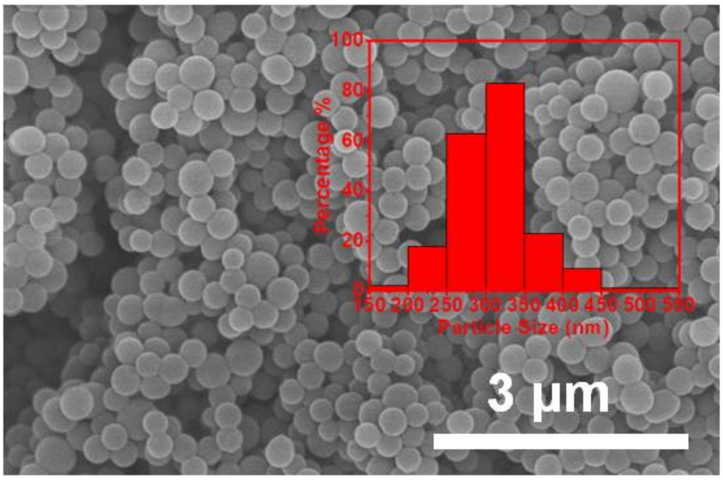

Figure 3. SEM image of PPy nanospheres, and the inset is the corresponding statistical data on the diameters of PPy nanospheres.

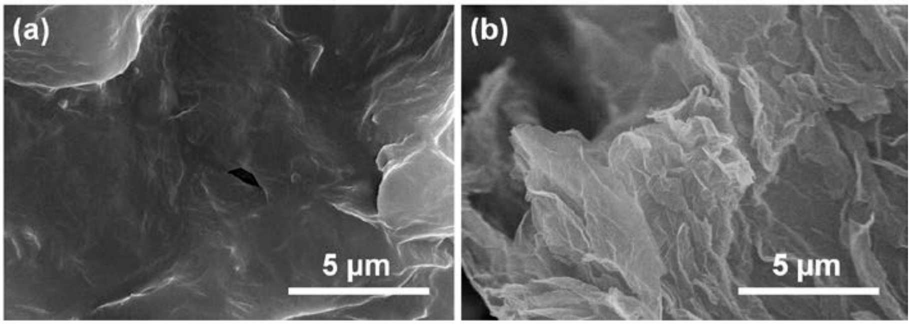

Figure S4. SEM images of commercial GO (a) and as-prepared rGO (b).

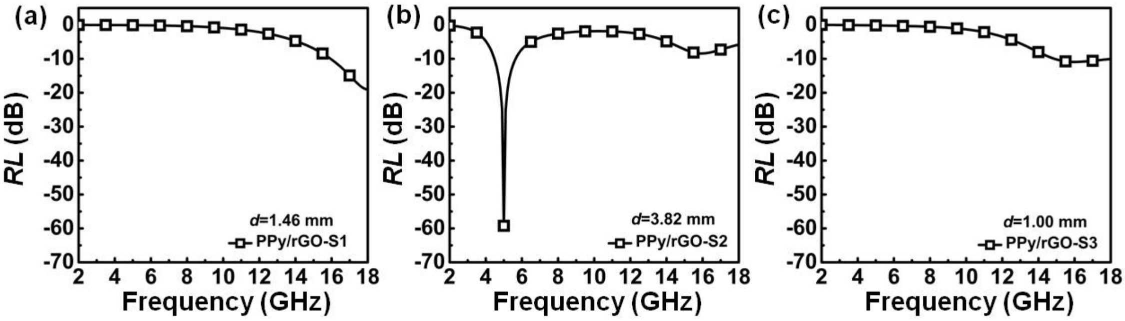

Figure S5. *RL* values of PPy/rGO-S1, PPy/rGO-S2, PPy/rGO-S3 with absorber thicknesses of 1.46 mm, 3.82 mm, and 1.00 mm, respectively.

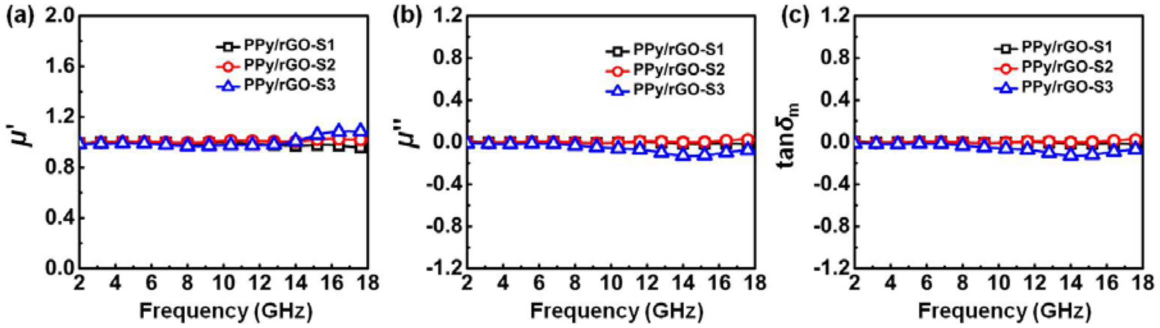

Figure S6. Real parts μ′ (a) and imaginary parts μ″ (b) of complex permeability of PPy/rGO-S1, PPy/rGO-S2, and PPy/rGO-S3, and their corresponding magnetic dissipation factor (c) in the frequency range of 2.0-18.0 GHz.
